# ENACT: a protocol for a randomised placebo-controlled trial investigating the efficacy and mechanisms of action of adjunctive *N*-acetylcysteine for first-episode psychosis

**DOI:** 10.1186/s13063-019-3786-5

**Published:** 2019-11-28

**Authors:** S. M. Cotton, M. Berk, A. Watson, S. Wood, K. Allott, C. F. Bartholomeusz, C. C. Bortolasci, K. Walder, B. O’Donoghue, O. M. Dean, A. Chanen, G. P. Amminger, P. D. McGorry, A. Burnside, J. Uren, A. Ratheesh, S. Dodd

**Affiliations:** 1 Orygen the National Centre of Excellence in Youth Mental Health, Centre for Youth Mental Health, University of Melbourne, Locked Bag 10 (35 Poplar Road), Parkville, VIC 3052 Australia; 2 Centre for Youth Mental Health, The University of Melbourne, Melbourne, VIC Australia; 3 Institute for Mental and Physical Health and Clinical Translation (IMPACT), Deakin University, School of Medicine, Geelong, VIC Australia; 4 The Department of Psychiatry and the Florey Institute for Neuroscience and Mental Health, The University of Melbourne, Melbourne, VIC Australia; 5 School of Psychology, University of Birmingham, Edgbaston, UK; 6 Centre for Molecular and Medical Research, School of Medicine, Deakin University, Geelong, VIC Australia

**Keywords:** First-episode psychosis, Psychotic disorders, Neuroprotective agents, Treatment, Outcome, *N*-acetylcysteine, Oxidative stress, Nitrosative stress, Inflammation, Glutathione, Schizophrenia, Depression, Bipolar disorder

## Abstract

**Background:**

First-episode psychosis (FEP) may lead to a progressive, potentially disabling and lifelong chronic illness; however, evidence suggests that the illness course can be improved if appropriate treatments are given at the early stages. Nonetheless, the efficacy of antipsychotic medications is suboptimal, particularly for negative and cognitive symptoms, and more efficacious and benign treatments are needed. Previous studies have shown that the antioxidant amino acid *N*-acetylcysteine (NAC) reduces negative symptoms and improves functioning in chronic schizophrenia and bipolar disorder. Research is scarce as to whether NAC is beneficial earlier in the course of illness. The primary aim of this study is to determine the efficacy of treatment with adjunctive NAC (2 g/day for 26 weeks) compared with placebo to improve psychiatric symptoms in young people experiencing FEP. Secondary aims are to explore the neurobiological mechanisms underpinning NAC and how they relate to various clinical and functional outcomes at 26- and 52-week follow-ups.

**Methods/design:**

ENACT is a 26-week, randomised controlled trial of adjunctive NAC versus placebo, with a 26-week non-treatment follow-up period, for FEP. We will be recruiting 162 young people aged 15–25 years who have recently presented to, and are being treated at, the Early Psychosis Prevention and Intervention Centre, Melbourne, Australia. The primary outcome is the Total Score on the Positive and Negative Syndrome Scale which will be administered at baseline, and weeks 4, 8, 12, 26 (primary endpoint), and 52 (end of study). Secondary outcomes include: symptomatology, functioning, quality of life, neurocognition, blood-derived measures of: inflammation, oxidative and nitrosative stress, and magnetic resonance spectroscopy measures of glutathione concentration.

**Discussion:**

Targeted drug development for FEP to date has generally not involved the exploration of neuroprotective agents. This study has the potential to offer a new, safe, and efficacious treatment for people with FEP, leading to better treatment outcomes. Additionally, the neuroprotective dimension of this study may lead to a better long-term prognosis for people with FEP. It has the potential to uncover a novel treatment that targets the neurobiological mechanisms of FEP and, if successful, will be a major advance for psychiatry.

**Trial registration:**

Australian New Zealand Clinical Trials Registry, ID: ACTRN12618000413224. Registered on 21 March 2018.

## Background

Psychotic disorders predominantly emerge in adolescence or young adulthood and are among the most burdensome and costly illnesses worldwide [1]. They cause significant distress for the young person and disrupt the attainment of vocational and educational goals, social relationships and identity formation [2]. There is some evidence that psychotic disorders are neuroprogressive, based on evidence of morphological brain changes, decline in functioning, treatment resistance, and increasing susceptibility for relapse with disease progression [3]. A first episode of psychosis (FEP) may thus foreshadow a potentially disabling and lifelong chronic illness. There is, however, provisional evidence to suggest that the course of this life-altering condition can be diminished if appropriate treatments are given in the early stages of illness [3] and that therapies may have greater efficacy if given earlier in the illness course [4].

The goals of treatment for FEP are to minimise the duration of psychosis, stabilise and remit symptomatology, and prevent relapse [5]. Second-generation antipsychotics (SGAs) are often used as the first line of treatment for FEP; however, the benefits of SGAs can be tempered by: (1) FEP patients being particularly sensitive to treatment side-effects such as weight gain; (2) low medication adherence with rates around 33–50% at 6–12 months [6]; (3) treatment resistance and persistent psychotic symptoms occurring in about 20% of patients; and (4) depression and anxiety following the incipient episode which is reported in more than 50% of patients [7]. While SGAs can reduce positive psychotic symptoms [8], they are generally not effective in reducing other illness features such as negative and cognitive symptoms [5], and their usefulness for preventing or impeding illness progression has not been established. Generally, SGAs block dopamine pathways; however, the molecular pathology underlying psychotic disorders is likely to be complex, and several pathophysiological mechanisms have been identified including dopaminergic dysregulation, disturbed glutamatergic neurotransmission, inflammation and oxidative stress [8].

There is a growing body of evidence from biomarker, genetic and epidemiological studies that inflammation and an aberrant immune response may underpin the pathogenesis and illness course of schizophrenia and psychosis [9]. Exposure to prenatal maternal immune activation increases the risk of offspring developing schizophrenia later in life [10, 11]. Individuals who have an autoimmune disease or any history of hospitalisation with infection are also at greater risk of developing schizophrenia [12, 13]. Microglia cells, which are key to the immune response in the central nervous system, are found in higher density in individuals with schizophrenia [9, 14–16] and in both ultra-high-risk individuals [17] and those with FEP [18]. However, not all studies have found differences between healthy controls and patients with FEP [19] or chronic schizophrenia [20].

Anomalies in peripheral inflammatory markers have also been seen from the FEP, during relapse, and later in the illness course [21, 22]. Meta-analytic findings suggest that there could be both trait and state peripheral inflammatory markers in those at the first episode and in those who have had acute relapses with schizophrenia [21]. Furthermore, such changes in peripheral inflammatory markers have been associated with severity of the psychopathology and neurocognitive impairments in FEP [22, 23]. Findings from a meta-analysis of cytokine function in FEP indicate that medication-naïve patients have significantly increased serum pro-inflammatory cytokine levels, including elevated interleukin-1β, soluble interleukin-2 receptor, interleukin-6 and tumor necrosis factor, relative to healthy controls [24] suggesting that inflammation is present at the earliest stages of the illness.

Apart from abnormalities in immune-inflammatory markers, there is evidence of changes in the oxidative and nitrosative stress pathways in both the first episode [24] and the more chronic phases of psychosis [25]. Abnormalities across a range of oxidative stress markers have been frequently reported in schizophrenia [26, 27]. Proton magnetic resonance spectroscopy (MRS) studies have provided in vivo evidence of altered glutamate and compromised glutathione (GSH) levels in the prefrontal cortex of patients with chronic schizophrenia [28–31]. Genetic associations with oxidative pathway genes including the *glutathione-S-transferase* gene are associated with schizophrenia and phenotypes have been explored for therapeutic intervention [32]. Findings in FEP have been more variable. A recent meta-analysis reported no significant differences between early onset FEP patients and healthy controls for six oxidative markers including catalase, GSH, glutathione peroxidase (GPx), superoxide dismutase, total antioxidant status and deoxyribonucleic acid (DNA) oxidative damage [30]. However, the authors outline differences in methodology leading to heterogeneity across studies that made interpretation of the data difficult [26]. The meta-analyses did support relationships between oxidative markers and clinical, cognitive and neurobiological outcomes, suggesting that increased oxidative stress may lead to poorer long-term outcomes in this population [30]. Given the oxidative imbalances reported in chronic schizophrenia, it is clear that there is a disturbance in these pathways that may be amenable to early intervention. Therapeutic targeting of oxidative and nitrosative pathways early may provide a neuroprotective effect that prevents the development of reactive oxygen species (ROS) imbalances in conjunction with remediated clinical episodes.

Schizophrenia and psychotic disorders are likely to result from multiple aetiologies. Current treatments, such as SGAs focus on regulating dopaminergic pathways with little effect on other glutamatergic pathways, inflammation or oxidative stress. This may explain why outcomes for individuals with psychotic disorders have remained largely unchanged in terms of rates of mortality, morbidity and disability [33]. Thus, there is an urgent need for novel therapies that may target multiple pathways and improve outcomes from the incipient psychotic episode.

*N*-acetylcysteine (NAC) has putative neuroprotective properties that act against neurotoxic effects of the disease processes in psychotic disorders. In particular, NAC impacts glutamate [34], improves mitochondrial dysfunction and apoptosis in mouse models of schizophrenia [35], reduces oxidative stress and inflammation and enhances neurogenesis [36] – but it is uncertain which mechanisms are operative or more important. Our group has previously shown that NAC was effective for the treatment of negative symptoms in schizophrenia [37], a finding independently replicated [38]. NAC is also efficacious for mood symptoms in bipolar disorder and depression [39], substance abuse, such as alcohol [40] and cannabis [41], and cognitive symptoms in schizophrenia [42].

There is only one study of NAC in the early stages of psychosis (less than 12 months of treatment for psychosis [43];). In this small, two-centre trial (Lausanne, Switzerland and Boston, USA), 63 participants were randomised to receive either an effervescent NAC tablet (2700 mg/day total NAC) or placebo for 6 months. At 6 months, there were no between-group differences on the primary outcome measure of Positive and Negative Syndrome Scale Negative Subscale (PANSS-), or on secondary outcomes such as positive symptoms, general symptomatology, or functional outcomes. There were, however, improvements in neurocognition (verbal fluency and processing speed) and redox markers including GSH in the medial prefrontal cortex and in blood cells seen in the NAC group. In subgroup analyses they found that PANSS Positive Symptom Subscale scores reduced after 2 months of NAC treatment in those individuals who had elevated blood-cell levels of GPx of > 22.3 U/gHb. This study included a small sample size and, therefore, lacked power for the primary analyses, the magnetic resonance spectroscopy (MRS, 10–12 per group) and the subgroup analyses. Modest baseline levels of negative symptoms restricted the potential for treatment change. Further examination of the impact of NAC on changes in cognition, neurochemistry and clinical symptoms in a larger cohort is required.

The primary aim of this randomised controlled trial (RCT) is to determine the efficacy of 26 weeks of adjunctive NAC (2 g/qds) compared to placebo as a useful treatment of psychiatric symptoms in young people (15–25 years of age, inclusive) who experience FEP. The trial agent will be adjunctive to treatment as usual (TAU) at a specialist early intervention service. A secondary aim is to explore how the multiple neurobiological mechanisms underpinning 2 g/qds NAC relate to clinical and functional outcomes following 26 weeks of adjunctive treatment with NAC, and at 52-week follow-up. Another secondary aim is to examine the relationships between gene expression and treatment response and/or medication side-effects.

The primary hypothesis is that 2 g/qds of NAC will be associated with lower total symptom severity (defined by the PANSS_T_ score) than placebo at the primary endpoint of 26 weeks. Secondary hypotheses are for the superiority of 2 g/qds NAC compared to placebo at the primary endpoint of 26 weeks and at the secondary endpoint of 52-week follow-up in terms of: (1) greater reductions in positive, negative, general and depressive symptomatology; (2) interviewer and participant ratings of clinical improvement; (3) improved global functioning and quality of life (QoL); (4) better neurocognitive functioning; (5) changes in blood-derived measures of inflammatory, oxidative and nitrosative stress; and (6) increases in brain-derived (MRS) measures of GSH concentration, and these increases will be positively correlated with changes in functional and structural connectivity of brain networks.

## Methods/design

### Study design and ethical approval

The study design is a 26-week, parallel-group, triple-blind, randomised, placebo-controlled study in patients with a FEP, allocated to receive either 2 g/qds active NAC or matched placebo as adjunctive to TAU in a specialised early intervention service. Equal numbers of active and placebo groups will be recruited (ratio 1:1). The primary endpoint is end of treatment at 26 weeks; with a post-discontinuation follow-up at 52 weeks.

Institutional ethics and governance approval was obtained from the Melbourne Health Human Research Ethics Committee (HREC 2017.145). The study Sponsor is Orygen, The National Centre of Excellence in Youth Mental Health (referred to as Orygen). The Sponsor will not be involved in study design; collection, analysis or interpretation of data; writing of the report; and will not participate in the decision to submit the report for publication. A steering committee comprising the principal investigator, coordinating chief investigator, associate investigators, project manager, study monitor and other team members will meet on a regular basis to oversee the conduct of the trial.

The trial will be conducted in accordance with Good Clinical Practice (GCP [44];) guidelines and the Declaration of Helsinki [45]. The protocol has been developed in line with GCP and the Standard Protocol Items: Recommendations for Interventional Trials (SPIRIT) guidelines [46]. A SPIRIT Checklist is provided as an Additional file 1. The study is registered with the Australian and New Zealand Clinical Trials Registry (ACTRN12618000413224). Any amendments to the protocol will result in notifications to relevant parties including ethics, the Sponsor and trial registry.

### Study setting

The study will take place at Orygen Youth Health (OYH) the Victorian State Government-funded public youth mental health service for western metropolitan Melbourne. The Early Psychosis Prevention and Intervention Centre (EPPIC), which is part of OYH, was the first Australian specialist early intervention service for FEP, established in 1992. EPPIC is located over two regions; Parkville and Sunshine. In 2002, international guidelines for early intervention were developed, in part, guided by the EPPIC model [47]. The core treatment elements include 18 months of: assertive case management; as necessary low-dose atypical antipsychotic medications; and evidence-based psychotherapy [47].

All participants will be recruited from the two EPPIC locations. Approximately 250–290 new clients are admitted to EPPIC each year. Previous studies targeting this population at OYH were able to achieve similar recruitment targets, with more stringent eligibility criteria (e.g. [48]). The recruitment forecast allows for the recruitment of one participant per week (average of four to five participants per month).

Individuals with psychotic disorders are notoriously difficult to recruit and retain in research; therefore, flexibility is essential. The study team has developed skills in engaging and retaining youth with psychotic disorders in research. A team-based approach is utilised, facilitated by tracking notes relating to all participant interactions and action plans in the Research Project Management System (RPMS), to ensure that clear and consistent messages and information are provided to the participants.

### Eligibility criteria

A participant will be considered eligible for inclusion in this study only if all of the following criteria apply: (1) aged 15 to 25 years (inclusive, at time of enrolment); (2) within their first 3 months of admission to EPPIC (defined as having signed consent within 3 months from date of first face-to-face outpatient visit with treating EPPIC clinician); (3) at least 2 weeks of stable use of their primary medication (e.g. antipsychotic medications); (4) if female*:* using effective contraception if sexually active; (5) capacity to consent to the study and comply with study procedures as ascertained by the research interviewer and treating team.

Participants who meet any of the any of following criteria will not be eligible for participation in this study: (1) previous distinct episode of psychosis with inter-episode recovery and recurrence, with the period of recovery lasting more than 6 months; (2) known or suspected clinically relevant systemic medical disorder, and/or recent gastrointestinal ulcers or renal stones; (3) female patients who are pregnant or lactating; (4) prior sensitivity or allergy to NAC; (5) currently taking > 250 mg NAC/day; or > 200 μg selenium/day (a 1-month washout period will be required if individuals who are currently taking these nutraceuticals would like to participate); (6) inability to comply with either the requirements of informed consent or the treatment protocol; and/or (7i) non-fluency in English.

There are additional exclusion criteria for the MRS component of the study. Participants will be excluded from MRS for the following reasons: (1) serious head injury; (2) seizures or history of epilepsy; (3) thyroid or neurological disorder; (4) claustrophobia or inability to tolerate length of time in the scanner; (5) any implanted metal (including certain types of cerebral clips); and (6) any implanted electronic device such as pacemaker, implantable cardioverter/defibrillator, insulin pump, implanted hearing device and/or neurostimulator.

A participant will be withdrawn if they: (1) cease taking their trial medication for seven consecutive days; (2) cease effective contraception or become pregnant; and/or (3) withdraw consent or develop serious adverse events associated with the study drug. Discontinuation due to adverse events could be either at the request of the participant or the discretion of the investigator.

### Study medication

Previous studies demonstrate that a dose of 2 g/day of NAC generally appears to be effective and well tolerated [49], and the majority of studies showing efficacy of adjunctive NAC treatment for psychotic and mood symptoms have utilised a daily dose of 2 g with no significant difference from placebo in side-effect profile [37–39, 50–53]. While several studies have administered NAC in a 1-g dose twice daily [37, 39, 50], NAC will be administered as a once daily dose of 2000 mg (2 × 1-g capsules to be taken with their usual medication at the same time each day) in the current study in order to improve treatment adherence. Two previous trials by our group utilised a 24-week treatment period [37, 39]. A similar treatment period will be used in the current study though the treatment period has been extended to 26 weeks. A 26-week post-treatment follow-up will be conducted to determine whether NAC improves outcomes over the longer term. This is especially salient since the trial of NAC in schizophrenia by Brier et al. [54] showed benefits that were most robust at 9 and 12 months.

To ensure blinding, placebo capsules will be carefully matched in appearance, flavour and packaging with the active treatment. The placebo will comprise encapsulated microcrystalline cellulose. As NAC has a distinctive odour, placebo capsules will be lightly dusted with NAC powder to help prevent unblinding.

The nature of the study population is such that participants will already be taking prescribed medications. Typical treatment at EPPIC comprises low-dose SGAs. Other medications may also be prescribed in this population, e.g. mood stabilisers, anticonvulsants, antidepressants, benzodiazepines, benztropine, propranolol and non-benzodiazepine sedatives, such as zopiclone and zolpidem, will be allowed. Antipsychotic medication dosing may change during the study period and this data will be captured. Antipsychotic dosing (risperidone equivalents) will be a covariate in the analysis of impact of NAC on the primary outcome.

Study medication will be dispensed at baseline, and follow-up visits at weeks 4, 8, 12 and 18.

### Measures

The schedule of assessments and endpoint measures are detailed in Table 1.

**Table 1:** Schedule of assessments

Visit number	1	2	3	4	5	6	7	8	9	10	11
Assessment	Screening	Baseline	Week 2^a^	Week 4	Week 8	Week 12	Week 18^a^	Week 26	Week 27^a^	Week 36^a^	Week 52
	Day − 21 to − 8	Day − 7 to 1	± 3 days	± 7 days	± 7 days	± 7 days	± 7 days	± 14 days	± 3 days	± 28 days	± 28 days
								(end of treatment)			(end of study)
Informed consent	x	–	–	–	–	–	–	–	–	–	–
Cohort characteristics and eligibility											
Demographics	x	–	–	–	–	–	–	x	–	–	x
Medical and psychiatric history	x	x	–	x	x	x	–	x	–	–	x
Inclusion/exclusion criteria for study	x	–	–	–	–	–	–	–	–	–	–
Eligibility screen for imaging	x	–	–	–	–	–	–	–	–	–	–
Pregnancy (urine)	x	–	–	–	–	–	–	–	–	–	–
SCID-5-RV	–	–	–	x	–	–	–	–	–	–	–
SCID-II PQ BPD screener/PID-5-BF	x	–	–	–	–	–	–	–	–	–	–
WASI-II	x	–	–	–	–	–	–	–	–	–	–
Randomisation	–	x	–	–	–	–	–	–	–	–	–
Primary outcome											
PANSS Total	–	x	–	x	x	x	–	x	–	–	x
Secondary outcomes											
Symptoms											
PANSS Positive, Negative and General	–	x	–	x	x	x	–	x	–	–	x
MADRS	–	x	–	x	x	x	–	x	–	–	x
CGI-S	–	x	–	x	x	x	–	x	–	–	x
CGI-I/PGI-I	–	–	–	x	x	x	–	x	–	–	x
Functioning and quality of life											
SOFAS	–	x	–	x	x	x	–	x	–	–	x
SDS	–	x	–	x	x	x	–	x	–	–	x
AQoL-8D	–	x	–	x	x	x	–	x	–	–	x
SIMI-LE	–	x	–	–	–	–	–	x	–	–	–
SIMPAQ	–	x	–	–	–	x	–	x	–	–	x
Neurocognition and imaging											
CogState	x	x	–	–	–	–	–	x	–	–	x
SDMT	–	x	–	–	–	–	–	x	–	–	x
Digit Span	–	x	–	–	–	–	–	x	–	–	x
RAVLT	–	x	–	–	–	–	–	x	–	–	x
Neuroimaging^b^	–	x	–	–	–	–	–	x	–	–	–
Substance use											
ASSIST	–	x	–	x	x	x	–	x	–	–	x
Research bloods											
Biomarkers and mRNA^c^	–	x	–	–	–	–	–	x	–	–	x
Clinical management											
Physical health assessments	–	x	–	x	x	x	–	x	–	–	x
Concomitant medications review	x	x	x	x	x	x	x	x	x	x	x
SAS	–	x	–	x	x	x	–	x	–	–	x
BARS	–	x	–	x	x	x	–	x	–	–	x
Adverse events (side-effects)	–	x	x	x	x	x	x	x	x	–	–
Treatment adherence (MARS)	–	–	x	x	x	x	x	x	–	–	–

^a^Assessment completed by telephone

^b^Imaging protocol includes functional magnetic resonance imaging (MRI) and magnetic resonance spectroscopy (MRS)

^c^Research bloods including blood-derived measures of inflammatory, oxidative and nitrosative stress, and gene expression (if consent provided)

Note: *AQoL-8D* Assessment of Quality of Life – 8 Dimensions, *ASSIST* Alcohol, Smoking and Substance Use Involvement Screening Test, *BARS* Barnes Akathisia Rating Scale, *CGI-I* Clinical Global Impressions – Improvement, *CGI-S* Clinical Global Impressions – Severity of Illness, *MADRS* Montgomery-Asberg Depression Rating Scale, *MARS* Medication Adherence Scale, *mRNA* messenger ribonucleic acid*, PGI-I* Patient Global Impressions – Improvement, *PID-5* Personality Inventory for DSM-5, *PANSS* Positive and Negative Symptom Scale, *RAVLT* Rey Auditory Verbal Learning Test, SDS, Sheehan Disability Scale, *SIMI-LE* Social Inclusion for People with Mental Illness – Long Edition, *SIMPAQ* Simple Physical Activity Questionnaire, *SAS* Simpson-Angus Scale, *SOFAS* Social and Occupational Functioning Scale, *SCID-5* Structured Clinical Interview for DSM-5, *SCID-II PQ BPD* Structured Clinical Interview for DSM-IV Axis II Disorders Personality Questionnaire, *SDMT* Symbol Digit Modality Test, *WASI-II* Wechsler Abbreviated Scale of Intelligence – Second Edition

#### Primary outcome

The Positive and Negative Symptom Scale (PANSS) will be used to measure overall symptom severity PANSS_T_, and severity of positive (PANSS+), negative (PANSS-) and general (PANSS_G_) psychopathology symptoms [55]. The PANSS has sound psychometric ratings in terms of both validity and reliability [56]. It has been widely used as an outcome measure in clinical trials for psychotic disorders. In the current study, change from baseline to week 26 on the PANSS_T_ score is the primary outcome measure.

#### Secondary outcomes

Secondary outcomes will include symptomatology, functioning and QoL, neurocognition, neuroimaging, substance use and biological correlates.

##### Symptomatology

The PANSS+, PANSS- and PANSS_G_, subscales will be used to assess positive and negative psychotic symptoms, and general psychopathology, respectively. Depressive symptoms will be assessed using the Montgomery-Åsberg Depression Rating Scale (MADRS [57];). The Clinical Global Impressions – Severity of Illness (CGI-S; 59) is a brief interviewer-rated scale that measures illness severity. The Clinical Global Impressions – Improvement (CGI-I [58];) is also an interviewer-rated scale and indexes the patient’s improvement or worsening relative to the baseline. The Patient Global Impressions – Improvement (PGI-I [59];), which is commensurate with the CGI-I, will be used as a self-report measure of perceived change since commencing treatment.

##### Functioning and quality of life

The Social and Occupational Functional Assessment Scale (SOFAS [60];) will be used to assess level of social and occupational functioning and is not directly influenced by the overall severity of the individual’s psychological symptoms. The Sheehan Disability Scale (SDS [61];) is a brief self-report tool developed to assess functional impairment in three inter-related domains; work/school, social and family life.

The Simple Physical Activity Questionnaire (SIMPAQ [62];) will be used to measure physical activity over a 7-day period around various assessment time pints. The SIMPAQ is a brief 8-item interview covering time spent in bed, sitting or lying down, walking, exercise, sport and other activities.

Social inclusion will be measured using the self-report questionnaire Social Inclusion in Mental Illness – Long Edition (SIMI-LE [63];). The SIMI-LE covers five inter-related domains: (1) housing, neighbourhood and services, (2) relationships, activities and setbacks, (3) employment and education, (4) finances and (5) health and wellbeing.

The Assessment of Quality of Life – 8 dimensions (AQoL-8D [64];) will be used as a measure of health-related QoL. The AQoL-8D was selected to assess units of quality-adjusted life years, enabling the quantification of the economic benefit of treatment.

##### Neurocognition

Neurocognition will be assessed using a variety of standardised, computer-based and pencil-and-paper tasks. Intellectual functioning will be assessed using the two subtest version Wechsler Abbreviated Scale of Intelligence – Second Edition (WASI-II [65];). The Cogstate computerised assessment battery will include Detection, Identification and *n*-back (1-back and 2-back) tasks. The Detection test measures processing speed using a simple reaction-time paradigm, the Identification test measures attention and processing speed using a choice reaction time paradigm, and the *n*-back tests measure visual working memory. Cogstate has demonstrated test-retest reliability and responsiveness, small practice effects, no significant floor or ceiling effects, and is an ideal measure to assess changes in neurocognition over time. Pencil-and-paper tests include: Symbol Digit Modalities Test (SDMT [66];) as a measure of visual attention and processing speed; Digit Span [67], a subtest of the Wechsler Adult Intelligence Scale designed to assess auditory-verbal attention span and working memory; and, the Rey Auditory Verbal Learning Test (RAVLT [68];) for the assessment of verbal learning and memory.

##### Neuroimaging

Participants will complete a 1-h magnetic resonance imaging MRI session on a 3-T Siemens Magnetom Skyra scanner at The Melbourne Brain Centre, Heidelberg, at two visits: (1) baseline and (2) end week 26. We will acquire structural, spectroscopy (GSH) and functional resting state, as follows:

High-resolution structural data will be acquired with a T1-weighted magnetisation-prepared rapid-acquisition gradient echo (MPRAGE) volumetric sequence.

Brain GSH concentration will be measured using a single-voxel MEscher-GArwood Point-RESolved Spectroscopy (MEGA-PRESS) spectral-editing sequence. A single voxel will be placed in the left anterior cingulate. This region has the advantage of showing GSH reductions in schizophrenia [69], being a major hub region for brain connectivity, and being relatively easy to shim.

Functional resting state MRI data will be acquired in the axial plane using a T2*-weighted echo-planar imaging sequence. Data will be acquired for approximately 10 min, during which time the participant will be asked to keep their eyes open and focussed on a fixation cross in the middle of the screen, but to allow their minds to wander.

For all sequences except the functional resting state participants will be asked to lie still and relax, and will have the option of listening to music or watching a DVD. Following the scans, a radiologist will review all images to assess whether there is any abnormal pathology. They will complete a report for each scan session and send this to the study’s designated physician/registrar. In the rare event that further investigation is required, the treating physician/registrar will contact the participant and discuss the radiological findings and refer on as needed.

##### Biological correlates

Measurement of inflammatory, oxidative and nitrosative stress markers will provide evidence of the drivers of neuroprogression and clarify the neuroprotective mechanism of NAC [3]. Approximately 25 mL of blood will be taken at baseline, at the 26-week treatment endpoint, and at 52-weeks’ follow-up. Cytokines associated with acute and chronic inflammation will be assessed. Markers of oxidative and nitrosative stress will also be analysed. Other markers may also be investigated if evidence of their involvement in these processes is demonstrated.

Participants will also be invited to provide a further 2.5-mL sample of blood (using the PaxGene™ tube) for analysis of genetic variation, and to identify the relationship between gene expression and treatment response, or medication side-effects.

#### Other measures

##### Cohort characteristics

Demographic data (sex, gender, age, living arrangements, educational level and employment status), medical and physical history, and medications, will be collected at screening with items sourced from Orygen’s harmonised questionnaire battery [70].

##### Diagnosis

Diagnostic information will be obtained using a number of measures. The Structured Clinical Interview for DSM-5 Research Version (SCID-5-RV [71];) modules pertaining to mood, psychosis, substance use and anxiety will be administered [71]. Because of the potential phenomenological overlap and difficulties of differential diagnosis of personality disorders (especially borderline personality disorder, BPD) and FEP [72], all the BPD items of the Structured Clinical Interview for DSM-IV Axis II Disorders Personality Questionnaire (SCID-II PQ [73];) will be administered. The 25-item Personality Inventory for DSM-5 brief form (PID-5-BF [74];) will be administered for a dimensional assessment of personality.

##### Medication adherence

The 10-item Medication Adherence Rating Scale (MARS, [75]) will be used to determine compliance with antipsychotic and trial medications. Compliance with the trial medications will also be determined by using a monthly pill count and can also be delineated from MRS and blood biomarkers.

##### Substance use

The Alcohol, Smoking and Substance Involvement Screening Test (ASSIST [76];) is a self-report measure for detecting and managing substance use and related problems in primary and general medical care settings. An overview of recent consumption and associated problems is obtained.

##### Physical health measures

Physical health measurements include weight, height, waist circumference, blood pressure and heart rate, and will be assessed at baseline, 4 weeks, 8 weeks, 12 weeks, 26 weeks and 52 weeks. These measurements will be completed by trained study team members.

### Determination of sample size

Previously, moderate effects have been observed (Cohen’s *d* = 0.57) in those who received NAC on global symptom outcomes over a 24-week period [37]. We have elected to use a conservative estimate of effect of 0.45 (Cohen’s *d*) for between-group differences on the PANSS_T_ score. With power (1 − β) set at 0.80, alpha (α) set at .05, and a one-tailed test we would need a total sample size of 124 (62 per group). Allowing for attrition rate of 30%, we calculate that we would need a total sample size of 162 (81 per group).

### Assignment of interventions

#### Randomisation

Participant eligibility will be established before randomisation. Participants will be assigned randomly and consecutively in a 1:1 ratio to one of two interventions, NAC or placebo. The randomisation sequence will be based on a computer-generated, permutated, block randomisation scheme with stratification for site. The randomisation sequence will be computer-generated by a statistician who is independent of the day-to-day conduct of the trial. The allocation sequence will be concealed within the RPMS.

#### Blinding

This study involves a triple-blind protocol. The Investigators, the Sponsor monitor, the study’s biostatistician, research assistants, the clinical treating teams, the laboratory team and the participant will be blinded to whether the participant is receiving NAC or placebo for the duration of the trial. Unblinding will only be permitted in the case of a medical emergency when the appropriate management of the patient necessitates knowledge of the treatment randomisation. All cases of unblinding will be documented.

#### Enrolment, consent and retention

**Fig. 1:**
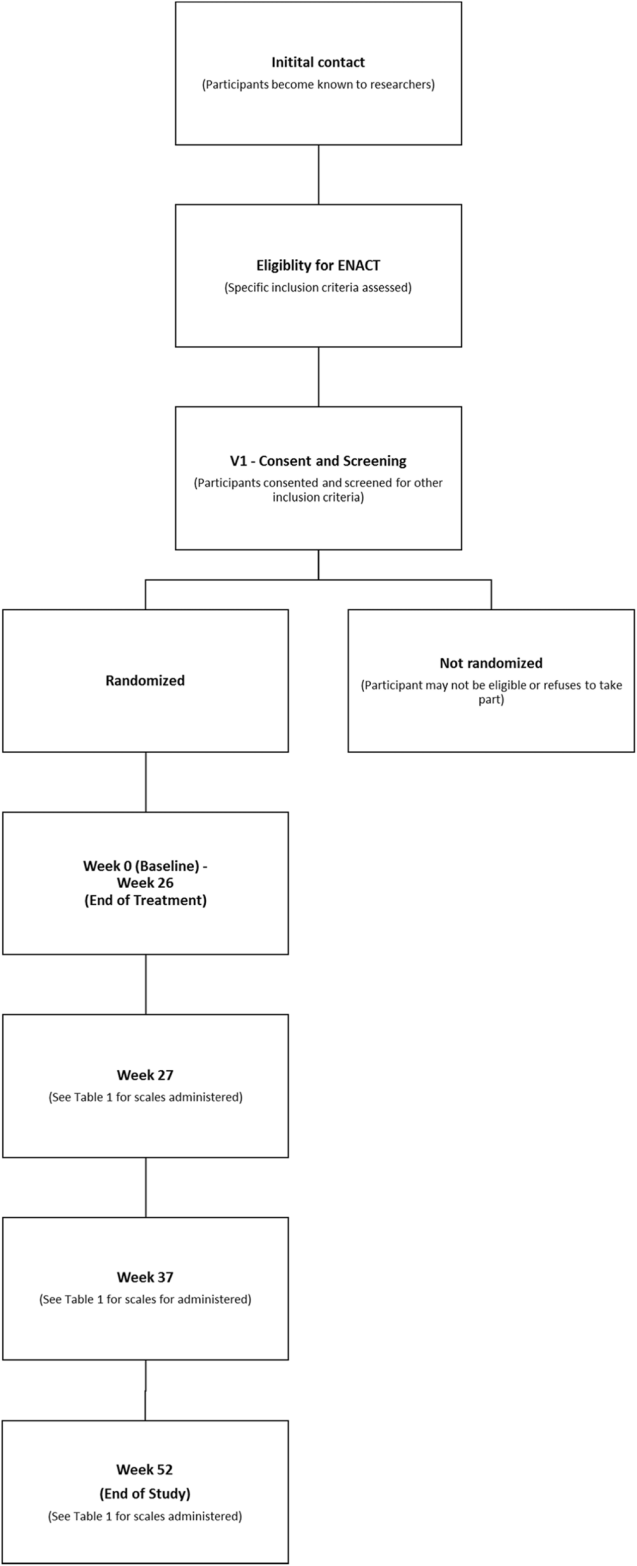
Flow chart of study design

Informed, written signed consent will be obtained by a trained research assistant from all participants aged 15 to 25 years inclusive. For those individuals less than 18 years of age, written informed consent will also be required from a parent or legal guardian. Optional consent will be sought for the use of de-identified information collected during this study for future research projects, and the storage and use of blood samples for this and associated studies. The Participant Information and Consent Form will be made available by the corresponding author upon request. Once consent is acquired, the initial screening assessment will be conducted to confirm eligibility. Prior to completion of the baseline assessment randomisation will occur. The participant flow through the study is detailed in Fig. 1. At the end of the study period, clinical care will continue to be provided to participants either through EPPIC (as determined by the EPPIC eligibility criteria) or the health service to which they have been discharged. Post-trial access to NAC will not be directly provided; however, as NAC is readily available as an over-the-counter supplement, a participant may continue with NAC treatment at the end of the trial treatment period under the guidance of their treating clinician, should they wish to do so.

### Data collection, management and analysis

The majority of assessments will be administered to participants in person via a semi-structured interview; however, assessments may also be conducted over the phone, or via online video services if required. Flexibility with timing and location of assessments (e.g. home visits, after school hours, etc.) will be essential to aid in participant retention. The study team will undergo extensive training prior to completing assessments, and inter-rater reliability between raters will be established. Consent will be sought from participants for audio recordings to be made of research visits to assist in the regular assessment of inter-rater reliability of various measures over the course of the study.

Each case report file (CRF) will be regularly monitored to ensure data quality, and de-identified data will be entered into an electronic CRF embedded within Orygen’s web-based Research Project Management System. The electronic CRF data will be audited to ensure data-entry accuracy.

Study protocol, statistical analysis plan, informed consent templates and de-identified participant data will be made available to investigators whose proposed use of the data has been approved by an independent review committee, for up to 3 years following publication.

Study findings will be presented at relevant conferences and published in peer-reviewed journals. A summary of the findings will be provided to participants on request.

#### Statistical methods

Statistical analyses will follow the International Conference on Harmonisation E9 statistical principles. Reporting of results will be done in accordance with Consolidated Standards of Reporting Trials (CONSORT) guidelines [77]. All participants who were randomised and who have at least one post-baseline observation will be included in a modified intent-to-treat analysis [78]. All attempts will be made to ensure that all participants have follow-up data even if they discontinue from the treatment. The primary efficacy analysis will assess average treatment group differences for the primary outcome measure (PANSS_T_) over the entire study period and will use a likelihood-based mixed-effects model, repeated-measures approach (MMRM). The MMRM model includes the fixed, categorical effects of treatment, visit, treatment-by-visit interaction, and Region (Parkville and Sunshine) will also be included as a factor in the model. Antipsychotic dosing may be also used as the covariate in the model. The MMRM includes all available data at each time point and is the preferred method of analysing clinical trial data in psychiatry as compared to more traditional repeated measures analysis of variance and analysis of covariance models. Planned comparisons will be done with the MMRM models to determine between-group differences in change in symptom measures from baseline to week 26. Differences between treatment groups in terms of secondary outcome measures will be examined using MMRM as per the primary outcome. Correlational analyses as well as multilevel modelling will be used to develop a better understanding of how changes in blood- and brain-derived biomarkers relate to symptoms, functioning and neurocognition. No interim analyses will be conducted.

Dynamic MEGA-PRESS MRS data will be corrected for frequency drift and edit-off and edit-on spectra combined to produce a GSH-edited spectrum. Edited data will be pre-processed and fitted using the TARQUIN analysis algorithm, with the resonance at 2.9 ppm used to determine the level of GSH. Matching water-reference data will also be collected and used to determine the absolute concentration of GSH. Resting-state functional imaging data will be analysed using the GraphVar toolbox and the Automated Anatomical Labelling atlas for parcellation. To delineate the default mode network (DMN) for each participant, functional connectivity (i.e. Pearson correlation) will be computed between the signal averaged over a spherical region of interest positioned at the medial prefrontal cortex and the signal at all other brain voxels. These functional connectivity maps will be *r*-to-*z* transformed and carried forward to a second-level statistical analysis to identify significant within- and between-group differences in DMN connectivity. Factorial models similar to the MMRM approach described above will be adopted to test for main effects and interactions using the appropriate combination of *F* and *t* tests. A cluster-based permutation approach will be used to identify significant differences satisfying a family wise error rate of .05. Lastly, betweenness-centrality will be calculated for the medial prefrontal cortex nodes and correlated with GSH concentration.

#### Monitoring

A Data Safety and Monitoring Committee (DSMC) will be established prior to commencement of recruitment. The DSMC will include a sponsor representative, a clinician, and a researcher with expertise in FEP, and an independent biostatistician. Data monitoring will be conducted by a clinical research associate.

#### Safety

A record will be made of any adverse event that arises during the trial. An adverse event will be defined as any unfavourable medical change that is accompanied by functional or clinical impairment, which may or may not be related to the study treatment. Any undesirable medical condition occurring from the time of signing consent (even if no study treatment or pharmaceutical product has been administered) will be considered to constitute an adverse event. In our NAC trials with schizophrenic, bipolar, and depressive populations, there have been no statistically significant differences between adverse events reported in the NAC or placebo groups, although numerically some gastrointestinal side-effects have been noted in the NAC groups.

## Discussion

Psychotic disorders are associated with substantial morbidity and mortality burden. FEP is a critical period where high-quality, stage-appropriate, proven treatments tailored to this unique population are most crucial. However, current pharmacological interventions have suboptimal therapeutic outcomes. Given that SGAs primarily block dopamine pathways, only one of many dysregulated pathways, it is not surprising that their efficacy is less than optimal. Molecular pathology underlying psychotic disorders is far more complex, and multiple operative systems have been identified apart from dopaminergic dysregulation, including disturbed glutamatergic neurotransmission, mitochondrial dysfunction, apoptosis, reduced neurogenesis and a pro-inflammatory status of the brain. NAC has broader effects with evidence that it impacts glutamate, reduces oxidative stress and inflammation, improves mitochondrial dysfunction, decreases apoptosis and enhances neurogenesis [79]. Given the evidence of the neuroprotective potential of NAC in individuals with enduring psychotic disorders, demonstrated clinical and cognitive improvements in schizophrenia, and changes in imaging markers of neuroprotection in other psychotic disorders, this study has the potential to support the development of a novel, safer and more efficacious treatment option for people with FEP. Investigating the effects of NAC on a broad range of secondary and exploratory outcomes, including both peripheral and cortical biomarkers of GSH and cognitive functioning in the early stages of illness, in addition to examining the neuroprotective properties of this agent, may uncover a mechanism that leads to new ways of treating these individuals. If successful, this will be a major advance for psychiatry leading to better long-term prognoses, as well as short-term symptom remission for patients with FEP.

### Trial status

The current ENACT protocol is Version 6.0 dated 15 April 2019. Recruitment for the trial has commenced. Recruitment for the trial commenced on the 13 December 2018. Recruitment is expected to be approximately completed by May 2022.

## Supplementary information

**Additional file 1:** Standard Protocol Items: Recommendations for Interventional Trials (SPIRIT) 2013 Checklist: recommended items to address in a clinical trial protocol and related documents*.

## Data Availability

Recruitment has commenced and data will not be released until the end of the trial, once the main analyses have been completed and the primary outcome manuscript published.

## Abbreviations

AQoL-8D: Assessment of Quality of Life – 8 dimensions
ASSIST: Alcohol, Smoking and Substance Involvement Screening Test
BPD: Bipolar disorder
CGI-I: Clinical Global Impressions – Improvement
CGI-S: Clinical Global Impressions – Severity of Illness
CONSORT: Consolidated Standards of Reporting Trials
CRF: Case report file
DMN: Default mode network
DSMC: Data Safety and Monitoring Committee
EPPIC: Early Psychosis Prevention and Intervention Centre
FEP: First-episode psychosis
GPx: Glutathione peroxidase
GSH: Glutathione
MADR: Montgomery-Åsberg Depression Rating Scale
MMRM: Mixed-effects models repeated measures
MRI: Magnetic resonance imaging
MRS: Magnetic resonance spectroscopy
NAC: *N*-acetylcysteine
NHMRC: National Health and Medical Research Council
Orygen: Orygen The National Centre of Excellence in Youth Mental Health
OYH: Orygen Youth Health
PANSS: Positive and Negative Syndrome Scale
PANSS_T_: Positive and Negative Syndrome Scale Total
PANSS+: Positive and Negative Syndrome Scale Positive Symptom Subscale
PANSS-: Positive and Negative Syndrome Scale Negative Symptom Subscale
PANSS_G_: Positive and Negative Syndrome Scale General Symptom Subscale
PGI-I: Patient Global Impressions – Improvement
PID-5-PF: Personality Inventory for DSM-5 brief form
RAVLT: Rey Auditory Verbal Learning Test
RCT: Randomised controlled trial
ROS: Reactive oxygen species
RPMS: Research Project Management System
QoL: Quality of life
SCID-5-RV: Structured Clinical Interview for DSM-5 Research Version
SCID-II PQ: Structured Clinical Interview for DSM-IV Axis II Disorders Personality Questionnaire
SDMT: Symbol Digit Modalities Test
SDS: Sheehan Disability Scale
SGAs: Second-generation antipsychotics
SIMPAQ: Simple Physical Activity Questionnaire
SIMI-LE: Social Inclusion in Mental Illness – Long Edition
SOD: Superoxide dismutase
SOFAS: Social and Occupational Functional Assessment Scale
TAU: Treatment as usual
WASI-II: Wechsler Abbreviated Scale of Intelligence – Second Edition
